# Relationships Between Diurnal Changes of Tongue Coating Microbiota and Intestinal Microbiota

**DOI:** 10.3389/fcimb.2022.813790

**Published:** 2022-03-31

**Authors:** Xiao-jing Guo, Tao Jiang, Xu-xiang Ma, Xiao-juan Hu, Jing-bin Huang, Long-tao Cui, Ji Cui, Xing-hua Yao, Yu-lin Shi, Jun Li, Zhi-ling Guo, Jin-di Lou, Meng-chen Liang, Hong-yuan Fu, Pei Yuan, Jia-yi Liu, Li-ping Tu, Jia-tuo Xu

**Affiliations:** ^1^ Basic Medical College, Shanghai University of Traditional Chinese Medicine (TCM), Shanghai, China; ^2^ Shanghai Collaborative Innovation Center of Health Service in Traditional Chinese Medicine (TCM), Shanghai University of Traditional Chinese Medicine (TCM), Shanghai, China

**Keywords:** humans, tongue coating microbiota, gut microbiota, diurnal variation, relationship

## Abstract

The oral cavity and the intestine are the main distribution locations of human digestive bacteria. Exploring the relationships between the tongue coating and gut microbiota, the influence of the diurnal variations of the tongue coating microbiota on the intestinal microbiota can provide a reference for the development of the disease diagnosis and monitoring, as well as the medication time. In this work, a total of 39 healthy college students were recruited. We collected their tongue coating microbiota which was collected before and after sleep and fecal microbiota. The diurnal variations of tongue coating microbiota are mainly manifested on the changes in diversity and relative abundance. There are commensal bacteria in the tongue coating and intestines, especially Prevotella which has the higher proportion in both sites. The relative abundance of Prevotella in the tongue coating before sleep has a positive correlation with intestinal Prevotella; the r is 0.322 (p < 0.05). Bacteroides in the intestine had the most bacteria associated with the tongue coating and had the highest correlation coefficient with Veillonella in the oral cavity, which was 0.468 (p < 0.01). These results suggest that the abundance of the same flora in the two sites may have a common change trend. The SourceTracker results show that the proportion of intestinal bacteria sourced from tongue coating is less than 1%. It indicates that oral flora is difficult to colonize in the intestine in healthy people. This will provide a reference for the study on the oral and intestinal microbiota in diseases.

## Introduction

The content of microbial cells and genes in the human body is huge (Gilbert et al., 2018). In the early life, microbiota begin to colonize in the human body. During or shortly after birth, the gut microbiota of newborns begin to establish, and they are affected by maternal and environmental microbes (Koenig et al., 2011). The activity and composition of the microbiota are affected by the genetic background, age, diet, and health of the host. In turn, the composition and activities of the microbiota will affect host metabolism and disease development (Ottman et al., 2012). The digestive tract has the highest density of bacteria in the human body. About 10^11^ bacterial cells flow from the oral cavity to the stomach every day. The oral cavity, pharynx, esophagus, and intestinal microbiota overlap partially, and the oral and intestinal microbiota has higher stability (Segata et al., 2012; Zhou et al., 2013).

The oral cavity may be a reservoir of potential enteric pathogens (Atarashi et al., 2017). Ingested oral bacteria have poor colonization ability in a healthy intestine, and the colonization of oral microbiota in the intestine can induce inflammatory diseases (Atarashi et al., 2017; Cao, 2017; Kato et al., 2018), such as rheumatoid arthritis (Zhang et al., 2015), IBD (Singhal et al., 2011; Said et al., 2014), and colorectal cancer (Koliarakis et al., 2019). The oral and intestinal microbiota can induce the generation of inflammatory immune cells which can migrate to the inflamed tissues. This has a major impact on the occurrence and/or progression of non-intestinal inflammatory diseases, such as cardiovascular disease, autoimmune encephalomyelitis, and arthritis (Cao, 2017). However, these studies only found that the intestinal flora associated with the disease was also distributed in the oral cavity and did not confirm whether these bacteria were derived from the ectopic colonization of the oral microbiota. Moreover, majority of studies had found that intestinal microbiota was related to the therapeutic effect of drugs (Alexander et al., 2017; Habtemariam, 2020; Wu et al., 2020), and it is also a possible target for the therapy of Chinese herbs (Tong et al., 2018; Liu et al., 2020). Clarifying the specific connection between the oral and intestinal microbiota is helpful for the prevention, diagnosis, and treatment of diseases.

However, the distribution of the microbiota at different sites in the oral cavity is different, but they have highly interactive connections in the phylogeny and the tongue is the center of these connections (Faust et al., 2012; Human Microbiome Project Consortium, 2012). Studies had found that tongue coating was a potential microbial pool in the oral microbiota (Tanner et al., 2002), and it could be used as a bacterial biomarker, such as chronic periodontitis (Galimanas et al., 2014), gastric cancer (Xu et al., 2019), and COVID-19 (Ren et al., 2021). Meanwhile, Chinese medicine believes that the tongue is the external manifestation of the digestive function, especially manifest on the tongue color and the thickness of tongue coating. Therefore, we choose tongue coating microbiota as the representative of oral bacteria in this study.

There are different methods used in studies on the relationships between the oral and intestinal microbiota. In microbiome studies, correlation analysis (Ren et al., 2021) and the occupied ratio of typical oral microbiota in the intestine (Atarashi et al., 2017) are often used. These methods can clarify the relationships and the role of the oral and intestinal microbiota in different diseases to a certain extent. However, the colonization relationship between oral and intestinal microbiota is still under research. Microbial source tracking is mainly used to determine the source of specific microorganisms (Liu et al., 2018). SourceTracker is one of the most effective methods for microbial traceability (Knights et al., 2011). It can evaluate all assignments of the sequence to all source samples, including unknown sources, and create a joint distribution of these distributions by Bayesian methods (McGhee et al., 2020). This method had been applied in determining the origin of neonatal oral microorganisms (Tuominen et al., 2019), the source of bacteria in the lung tissue of chronic obstructive pulmonary disease (Pragman et al., 2018), and the relationship between nasal microbes and environmental ventilation in hospitalized patients (Chen et al., 2019). It will help us clarify the ectopic colonization proportion of oral bacteria in the intestine of healthy people.

Besides, human microorganisms have their circadian rhythm changes and maintain the host’s circadian rhythm as an important factor (Thaiss et al., 2016; Nobs et al., 2019). The circadian clock controls various physiological, metabolic, and behavioral functions of the human body. It is also related to the body’s immune function (Curtis et al., 2014), virus replication (Ehlers et al., 2018), the degree of lung inflammation, and the response to bacterial infections (Gibbs et al., 2014). The physiological changes of the host are mostly related to the diurnal oscillations of microorganisms. The intestinal microbiota undergoes diurnal composition and functional oscillations, which can affect the host’s metabolic homeostasis. The circadian oscillation of the salivary microbiota enriched the functions of environmental response in the evening, as well as the metabolic functions of vitamin and fatty acid biosynthesis in the morning (Thaiss et al., 2016; Takayasu et al., 2017; Nobs et al., 2019). Studies have shown that there are obvious circadian changes in the oral microbiota, especially manifested on the distribution and the abundance of bacteria (Takayasu et al., 2017; Carlson-Jones et al., 2020). However, how the diurnal change in the diversity of tongue coating microbiota happens and whether there is a change in the connection between the tongue coating microbiota which are obtained at different times and the intestinal microbiota are not yet clear. In this study, we collected tongue coating microbiota at different times to observe the changes of the relationship between oral and intestinal flora. We used the SourceTracker method to clarify the ectopic colonization of oral flora in the intestine in healthy people. This will provide an important reference for the disease research in future.

## Materials and Methods

### Study Participants

We recruited healthy undergraduates and postgraduates from Shanghai University of Traditional Chinese Medicine in this study. The age was 18–25 years; physical health is defined as no systemic diseases (such as diabetes, HIV infection, or genetic diseases) and no type of ongoing benign and malignant diseases that may interfere with the evaluation of the research objectives; oral health is defined as no oral cavity diseases, such as leukoplakia, erythema, and oral lichen planus (OLP); and sleeping health is assessed using the Pittsburgh Sleep Quality Index (PSQI). Participants who used the topical antibiotics in the past 7 days, accepted antibiotic treatment in the past 3 months, and had a long history of smoking, alcohol abuse, and/or drug abuse were excluded. We also excluded the pregnant and lactating women and ensured all participants must meet the above requirements. We recorded the participants’ diet types (all the participants were omnivores), taste preferences, and the basic information, such as height, weight, waist circumference, hip circumference, and blood pressure. All participants signed informed consent which were approved by the ethics committee of Shuguang Hospital Affiliated to Shanghai University of TCM (2018-626-55-01).

### Sample Collection

We collected tongue coating microbiota at 20:00–21:00 and at 7:00–8:00 the next day. All participants were asked to eat regularly 1 week before sampling, have their mouth washed with water thoroughly at least 2 h before the evening collection, keep fasted, no gargling, and no brushing of teeth until the morning collection is finished. The sterile swab was used to scrape the tongue coating at the middle site of the tongue dorsum back and forth rotatingly at least 5 times. Each sterile swab was stored in a sterile and enzyme-free Eppendorf tube (Beijing Labgic Technology Co., Ltd.) and was immediately placed in an ice cube for transportation to a −80°C freezer within half an hour. The stool samples were collected by the participants themselves and brought with refrigerants to the researchers and transferred to the -80°C freezer immediately.

### DNA Extraction, PCR Amplification, and Sequencing

#### DNA Extraction

Total genomic DNA samples were extracted using the OMEGA Soil DNA Kit (M5635-02) (Omega Bio-Tek, Norcross, GA, USA), following the manufacturer’s instructions, and stored at -20°C prior to further analysis. The quantity and quality of extracted DNAs were measured using a NanoDrop ND-1000 spectrophotometer (Thermo Fisher Scientific, Waltham, MA, USA) and agarose gel electrophoresis, respectively.

#### 16S rRNA Gene Amplicon Sequencing

PCR amplification of the bacterial 16S rRNA gene V3–V4 region was performed using the forward primer 338F (5′-ACTCCTACGGGAGGCAGCA-3′) and the reverse primer 806R (5′-GGACTACHVGGGTWTCTAAT-3′). Sample-specific 7-bp barcodes were incorporated into the primers for multiplex sequencing. The PCR components contained 5 μl of buffer (5×), 0.25 μl of FastPfu DNA Polymerase (5 U/μl), 2 μl (2.5 mM) of dNTPs, 1 μl (10 µM) of each forward and reverse primer, 1 μl of DNA template, and 14.75 μl of ddH2O. Thermal cycling consisted of initial denaturation at 98°C for 5 min, followed by 25 cycles consisting of denaturation at 98°C for 30 s, annealing at 53°C for 30 s, and extension at 72°C for 45 s, with a final extension of 5 min at 72°C. PCR amplicons were purified with Vazyme VAHTSTM DNA Clean Beads (Vazyme, Nanjing, China) and quantified using the Quant-iT PicoGreen dsDNA Assay Kit (Invitrogen, Carlsbad, CA, USA). After the individual quantification step, amplicons were pooled in equal amounts, and paired-end 2 × 250-bp sequencing was performed using the Illumina NovaSeq platform with NovaSeq 6000 SP Reagent Kit (500 cycles) at Shanghai Personal Biotechnology Co., Ltd. (Shanghai, China). In addition, the nucleotide sequences of all samples were submitted to the National Center for Biotechnology Information Search database (NCBI) database (PRJNA 782768).

### Sequence Analysis

Microbiome bioinformatics were performed with QIIME2 2019.4 (Bolyen et al., 2019) with slight modification according to the official tutorials (https://docs.qiime 2.org/2019.4/tutorials/tutorials/). Briefly, raw sequence data were demultiplexed using the demux plugin followed by primer cutting with the cutadapt plugin (Martin, 2011). Sequences were then quality filtered, denoised, merged, and chimera removed using the DADA2 plugin (Callahan et al., 2016). Non-singleton amplicon sequence variants (ASVs) were aligned with mafft (Katoh et al., 2002) and used to construct a phylogeny with fasttree2 (Price et al., 2009). Alpha diversity metrics [Shannon (Shannon, 1948))], beta diversity metrics [unweighted UniFrac (Lozupone and Knight, 2005)], and Jaccard distance (Jaccard, 1908) were estimated using the diversity plugin with samples rarefied to 30,477 sequences per sample. Taxonomy was assigned to ASVs using the classify-sklearn naive Bayes taxonomy classifier in the feature-classifier plugin (Bokulich et al., 2018) against the HOMD database (Escapa et al., 2020).

### Bioinformatics and Statistical Analysis

Sequence data analyses were mainly performed using QIIME2 and R packages (v3.2.0). ASV-level alpha diversity indices and the Shannon diversity index were calculated using the ASV table in QIIME2 and visualized as box plots. The Kruskal–Wallis rank-sum test and Dunn’s test were used as *post-hoc* tests to verify the significance of the difference. ASV-level-ranked abundance curves were generated to compare the richness and evenness of ASVs among samples. Beta diversity analysis was performed to investigate the structural variation of microbial communities across samples using Jaccard metrics (Jaccard, 1908) and UniFrac distance metrics (Lozupone and Knight, 2005) and visualized *via* principal coordinate analysis (PCoA) hierarchical clustering (Ramette, 2007). The significance of differentiation of the microbiota structure among groups was assessed by PERMANOVA (permutational multivariate analysis of variance) (Brian et al., 2001) using QIIME2.The taxonomy compositions and abundances were visualized using MEGAN (Huson et al., 2011) and GraPhlAn (Asnicar et al., 2015). A Venn diagram was generated to visualize the shared and unique ASVs among samples or groups using R package “VennDiagram,” based on the occurrence of ASVs across samples/groups regardless of their relative abundance (Zaura et al., 2009). LEfSe (linear discriminant analysis effect size) was performed to detect differentially abundant taxa across groups using the default parameters (Segata et al., 2011). Random forest analysis was applied to discriminate the samples from different groups using QIIME2 with default settings (Breiman, 2001; Liaw and Wiener, 2001). Nested stratified k-fold cross validation was used for automated hyperparameter optimization and sample prediction. The number of k-fold cross-validations was set to 10. Co-occurrence network analysis was performed by SparCC analysis. The pseudocount value in SparCC was set to 10^-6^. The cutoff of correlation coefficients was determined as 70 through random matrix theory-based methods as implemented in R package RMThreshold. Based on the correlation coefficients, we constructed a co-occurrence network with nodes representing ASVs and edges representing correlations between these ASVs. The network was visualized using Cytoscape(Version 3.8.2). In order to clarify the relationships between the oral microbiota and the intestinal microbiota, we used SourceTracker (Knights et al., 2011) to determine the proportion of the intestinal microbiota which derived from the oral cavity. The Wilcoxon rank test (paired samples) was used to analyze the significance of microbial differences and visualized with GraphPad Prism (Version 8.0.1). Spearman’s correlation was used to analyze the relationship between the abundance of oral microbiota and intestinal microbiota by using IBM SPSS Statistics 26.0. p values ≤0.05 were considered significant.

## Results

### Characteristics of the Study Participants and Sequencing Results

We recruited 47 volunteers in total and analyzed the basic data and microbial samples of 39 volunteers after screening (**Supplementary Figure 1**). The age distribution of 39 volunteers was 21.35 ± 2.38 years, and their sleep quality was normal after PSQI assessment (**Supplementary Table 1**). 117 microbial samples (78 tongue coating samples and 39 stool samples) were collected for analysis, and a total of 8,855,246 sequences were detected. The average effective sequence amount for each sample was 71,399.57, the average high-quality sequence amount was 51,647.98, and the average sequence length was 420.6217.

### Circadian Oscillations of Human Tongue Coating Microbiome

**Figure 1:**
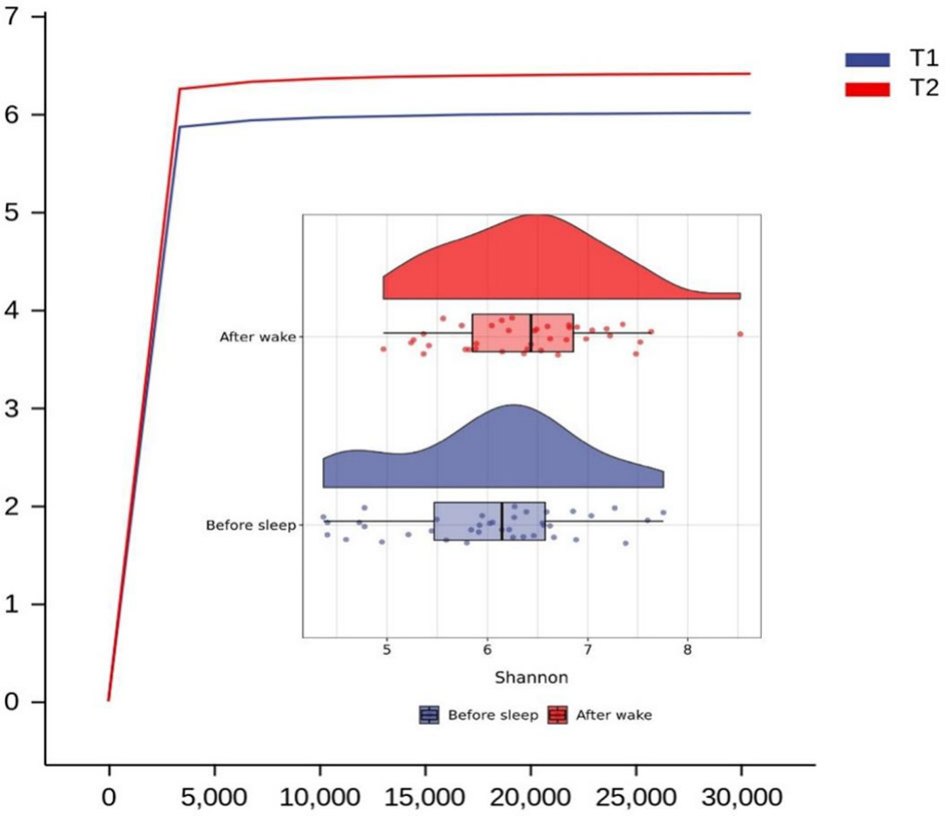
Rarefaction and Raincloud Plot of the tongue coating microbiota before and after sleep and the difference between them in Shannon diversity index (T1 and T2 are the tongue coating microbiota before and after sleep, respectively).

**Figure 2:**
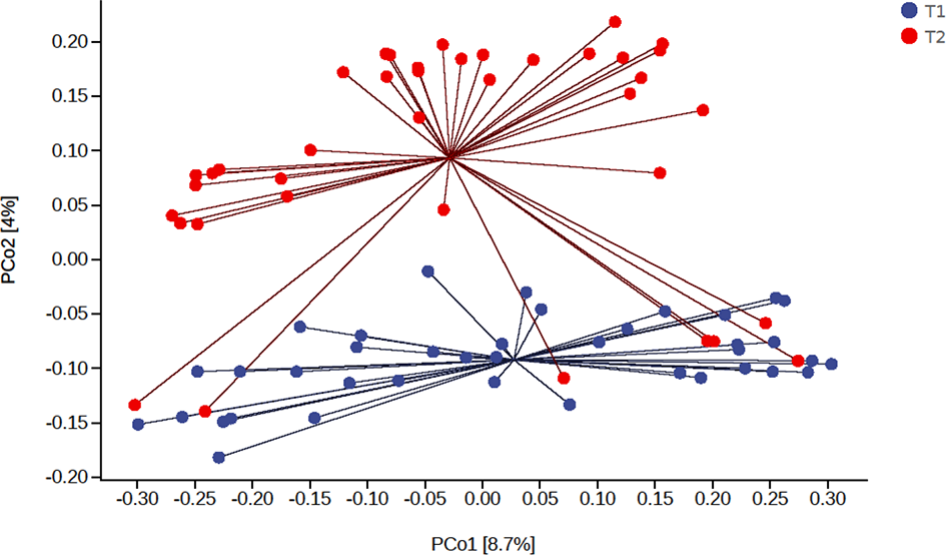
The principal coordinate analysis of the difference of the tongue coating microbiota before and after sleep (the percentage in the brackets of the coordinate axis represents the proportion of sample difference data (distance matrix) that can be explained by the corresponding coordinate axis).

We found that the main species of tongue coating microorganisms are Bacteroidetes, Firmicutes, Fusobacteria, Proteobacteria, and Actinobacteria at the phylum level. This was consistent with another study (Chen et al., 2021). The total ASV count of the tongue coating microbiota in the morning was higher than that before sleep (**Supplementary Figure 2**), while the absolute abundance of their shared ASV accounted for 97.05% and 95.84% in the total microbiota before and after sleep, respectively. This means that the main distribution of the tongue coating microbiota is stable. We used the Shannon index to compare the alpha diversity’s circadian changes of the tongue coating microbiota. The richness and evenness of the tongue coating microbiota in healthy people were higher in the morning, but there was no statistical difference (**Figure 1**). The principal coordinate analysis and Permanova were used to analyze the differences between groups, and we found that the distribution of tongue coating microbiota before and after sleep was different (p = 0.001, **Figure 2** and **Supplementary Table 2**). In the composition of the microbiota, the relative abundance of Prevotella, Porphyromonas, and Peptostreptococcus increased, and the relative abundance of Streptococcus, Actinomyces, Leptotrichia, etc., was decreased in the morning (**Supplementary Figures 3, 4** and **Supplementary Table 3**).

**Figure 3:**
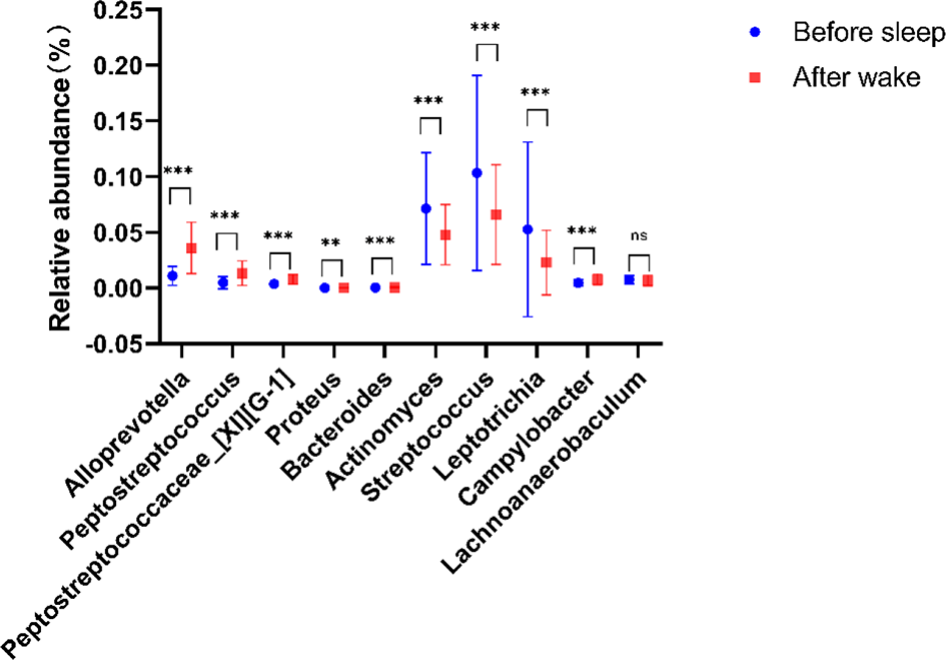
Compared with tongue coating microbiota before sleep, five genera were significantly enriched, while four genera were significantly reduced after waking up (**p < 0.01, ***p < 0.001, ns means p > 0.05).

To determine the main different species of the tongue coating microbiota before and after sleep, we used the random forest (10-fold cross-validations) to screen the main 10 bacterial genera and analyzed the significance of their relative abundance differences (**Supplementary Table 4**). At the genus level, the relative abundance of Alloprevotella, Peptostreptococcus, Peptostreptococcaceae_[XI][G-1], Bacteroides, and Campylobacter increased and the Proteus, Actinomyces, Streptococcus, Leptotrichia, and Lachnoanaerobaculum decreased in the morning (**Figure 3**). These suggested that there were circadian changes in the tongue coating microbiota, which are mainly manifested on the changes of the abundance, while the evenness and richness of the tongue coating microbiota had no significant difference and the main microbiota distribution was relatively stable.

**Figure 4:**
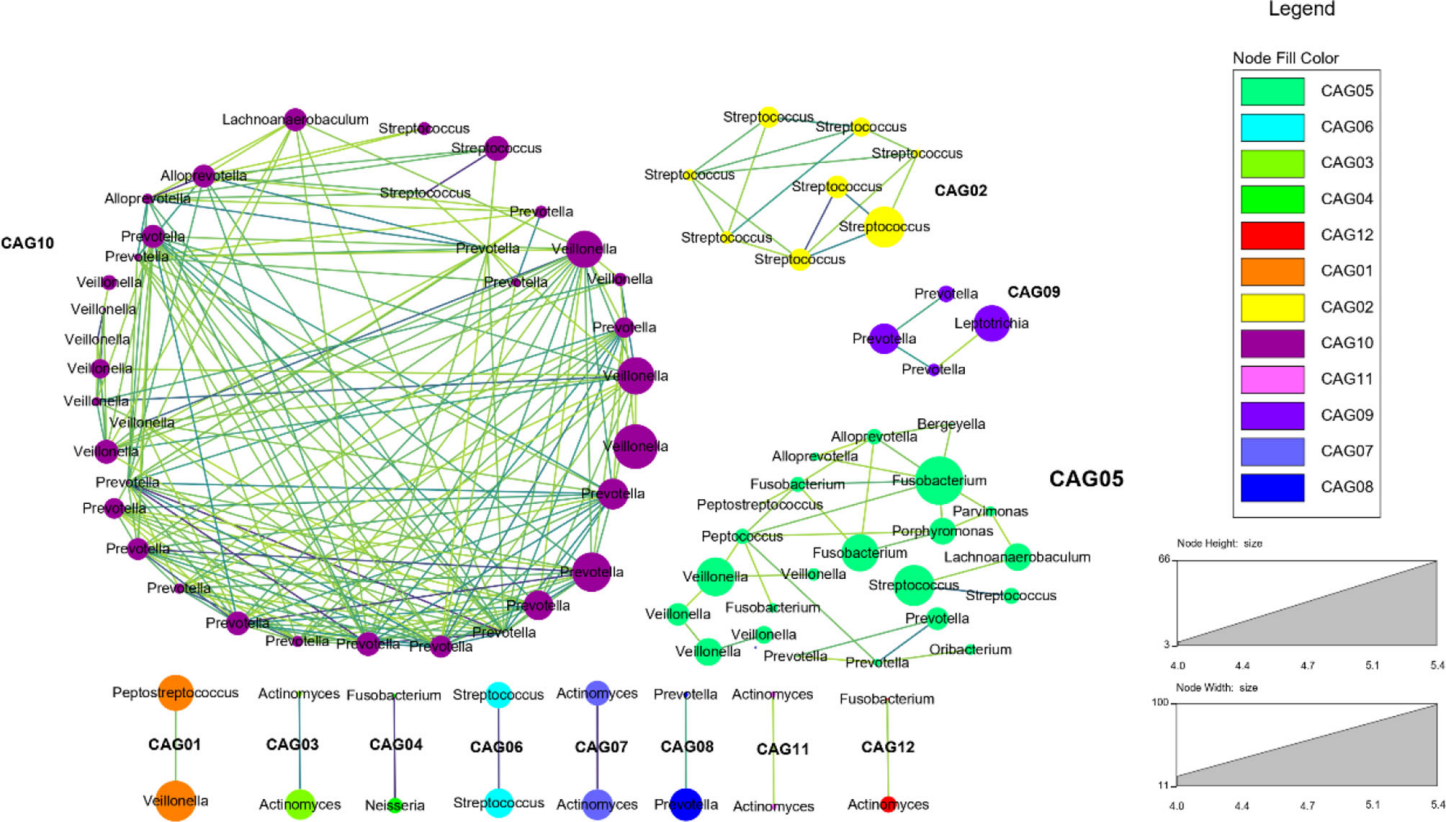
The co-abundance network of the tongue coating microbiota (at the genus level, different dot colors in the figure represent different modules, the larger the circle, the higher the abundance; the darker the color of the connecting line between the modules, the higher the correlation).

Meanwhile, we selected the bacteria which had a Spearman’s correlation ≥0.6 and p value ≤0.05 to construct the co-occurrence network. It could show the co-occurrence and co-exclusion relationships between tongue coating microbiota. We found that the tongue coating microbiota of healthy people could be clustered into 12 co-abundant groups (CAGs) (**Figure 4**). Among them, CAG10 contained the most bacteria, including Alloprevotella, Prevotella, Veillonella, Streptococcus, and Lachnoanaerobaculum (at the genus level). Their abundance changes were positively correlated. Studies have shown that only the bacterial community can degrade the complex substrates to benefit the growth of the entire community (Wade, 2013). These co-abundance relationships revealed the survival status of tongue coating microbiota.

### The Similarities and Differences in the Composition of Oral and Gut Microbiota

**Figure 5:**
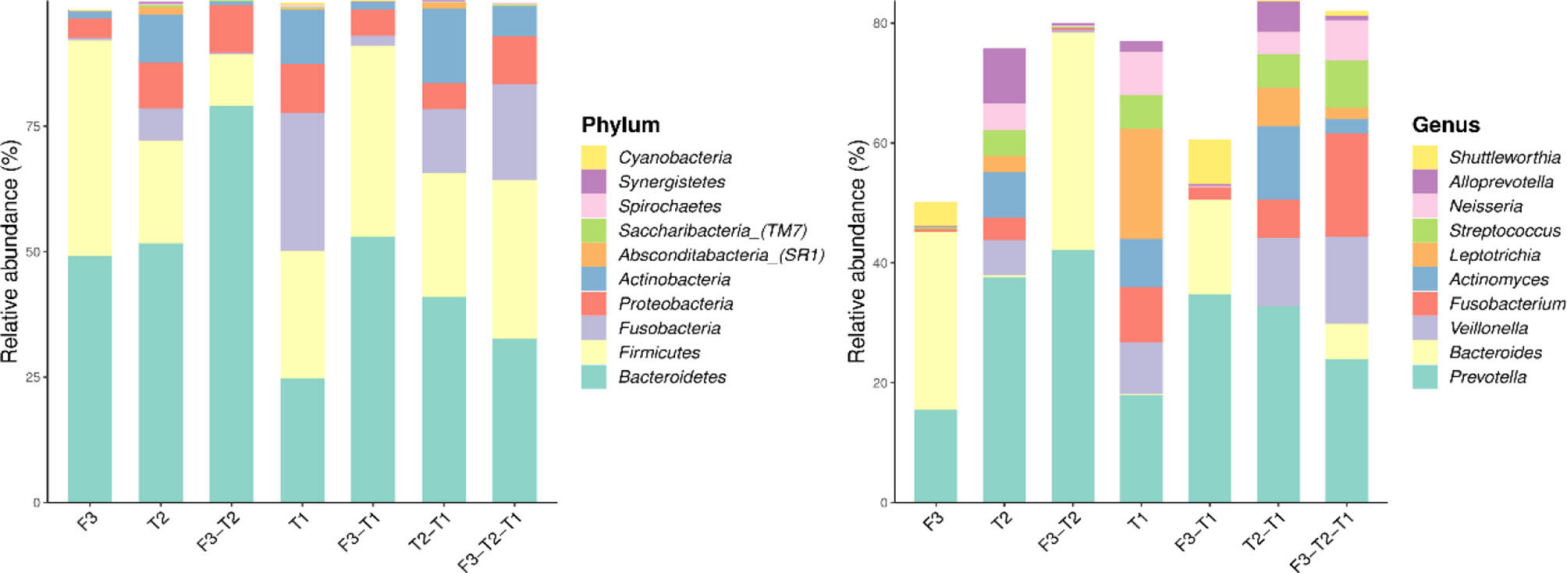
The common and unique ASV composition of intestinal and tongue coating microbiota (only show the top 10 in abundance, the left is at the phylum level, the right is at the genus level).

**Figure 6:**
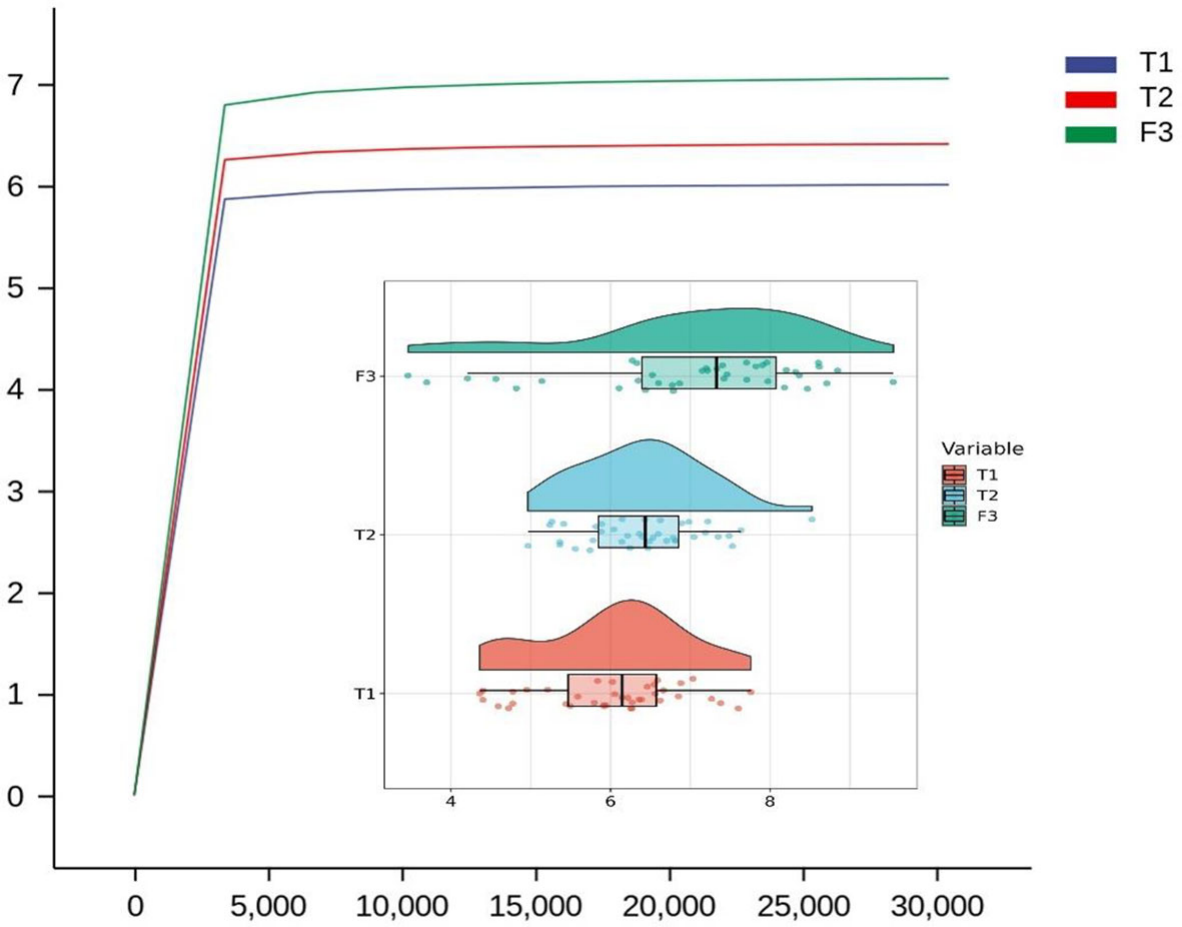
The Rarefaction Curve and Raincloud Plot of tongue coating and intestinal microbiota and the difference of Shannon index (T1 is the tongue coating microbiota before sleep, T2 is the tongue coating microbiota after waking up, and F3 is the intestinal microbiota).

**Figure 7:**
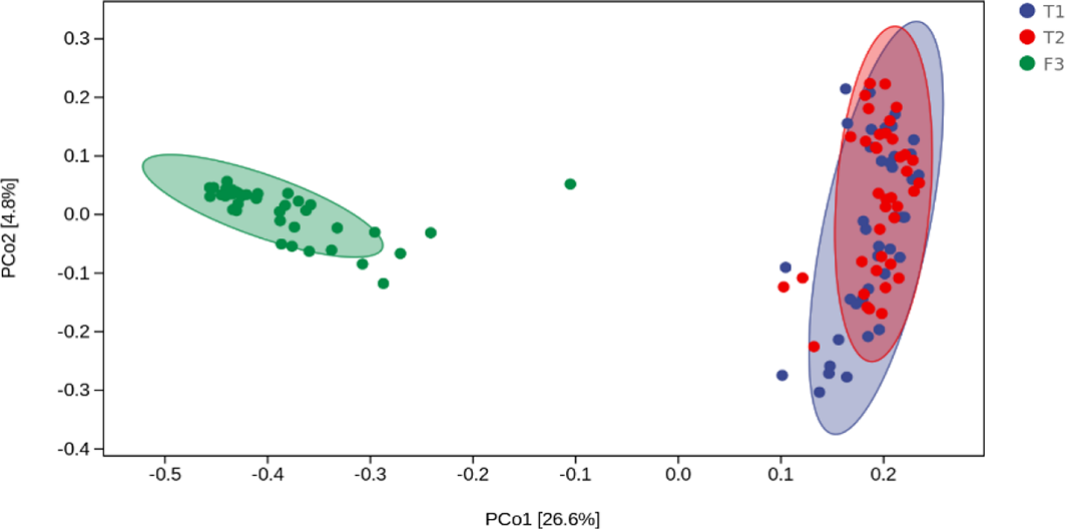
The principal coordinate analysis diagram of the composition of tongue coating and intestinal microbiota (the percentage in the brackets of the coordinate axis represents the proportion of sample difference data (distance matrix) that can be explained by the corresponding coordinate axis.).

**Figure 8:**
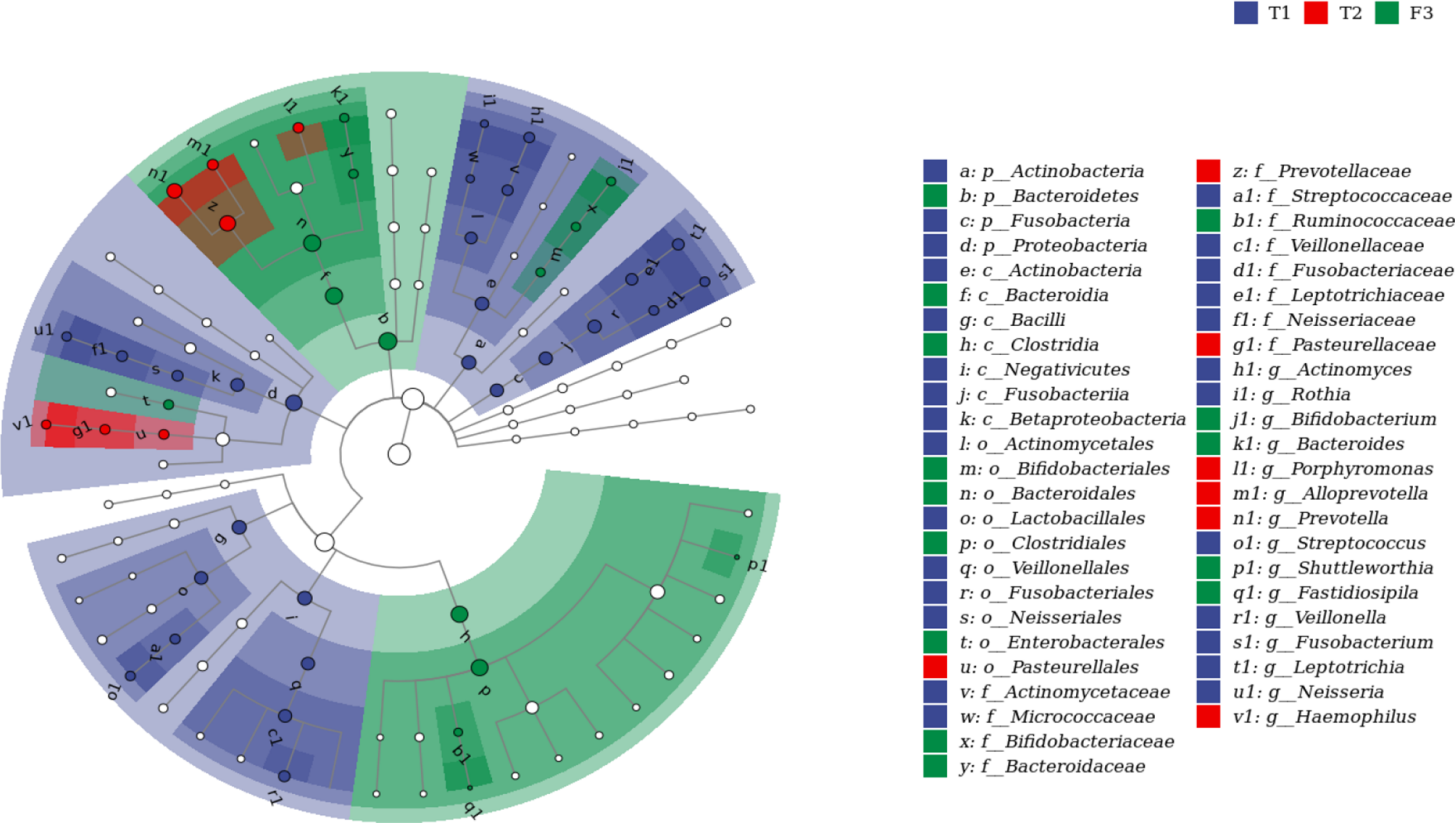
The LEFSE analysis diagram of tongue coating and intestinal microbiota (the top 50 genera with LDA value, and the LDA threshold is 3.87. From the inner circle to the outer circle is the classification hierarchical relationship of the main taxa (from phylum to genera). The node size corresponds to the average relative abundance of the taxa; the hollow nodes represent taxa with insignificant differences between groups, while nodes with other colors (such as green and red) indicate that these taxa exhibit significant differences between groups (p < 0.05), and the color represents a higher abundance in the grouping samples. The letters identify the names of taxa with significant differences between groups).

We analyzed the similarities and differences between the tongue coating microbiota and the intestinal microbiota of 39 volunteers to identify their associations. The number of ASV in the intestinal microbiota was significantly higher than that of the tongue coating. The proportion of the ASV that is shared by the tongue coating and intestine is 0.67%. Compared to the tongue coating microbiota at night, the number of ASV shared by the tongue coating and the intestine after waking up is greater (**Supplementary Figure 2**). After annotation, we found that the common microbiota between the tongue coating and the intestine are dominated by Bacteroidetes, Firmicutes, and Proteobacteria at the phylum level, and Prevotella and Bacteroides at the genus level (**Figure 5**). The results of diversity analysis showed that under the same sequencing depth, the abundance and diversity of intestinal microbiota were higher, comparing to tongue coating microbiota, and the difference was significant (p < 0.05) (**Figure 6**). The principal coordinate analysis method was used to analyze the difference between the intestinal and tongue coating microbiota, and the results showed that there was a significant difference in the community composition between the tongue coating and the intestine (unweighted UniFrac distance) (**Figure 7**). LEfSe analysis showed that the abundance of Bacteroidetes was higher in the intestine, while the abundance of Fusobacteria, Prevotellaceae, and Actinobacteria was higher in the tongue coating (p < 0.05) (**Figure 8**).

### The Relationships Between Oral and Intestinal Microbiota

In order to clarify the colonization of the oral microbiota in the intestine and the relationship between the distribution of the oral and intestinal microbiota in healthy people, we selected the ASV relative abundance greater than 1% as the typical microbiota of the human oral microbiota and intestine. In the oral microbiota, we selected 16 ASVs belonging to 6 genera before going to bed, 15 ASVs belonging to 7 genera after waking up. Among them, 5 typical bacterial genera were shared: Fusobacterium, Veillonella, Prevotella, Streptococcus and Neisseria. This suggested that the main species composition of the oral microbiota of healthy people was relatively stable.

**Figure 9:**
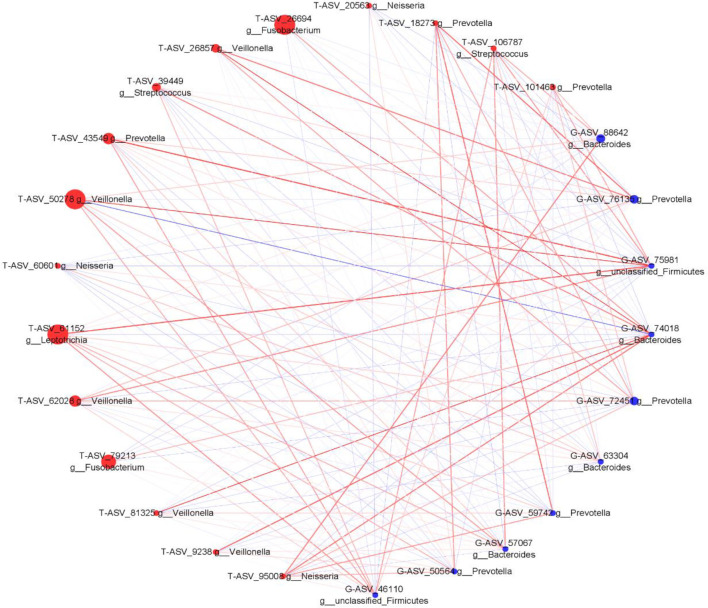
The associated network diagrams of typical tongue coating before sleep and typical intestinal microbiota. (red is the tongue coating microbiota, blue is the intestinal microbiota, the size of the circle represents the abundance of the flora, the larger the area of the circle, the higher the abundance; the red line represents the positive correlation, the blue line represents the negative correlation, the thicker the line, the higher the correlation coefficient).

**Figure 10:**
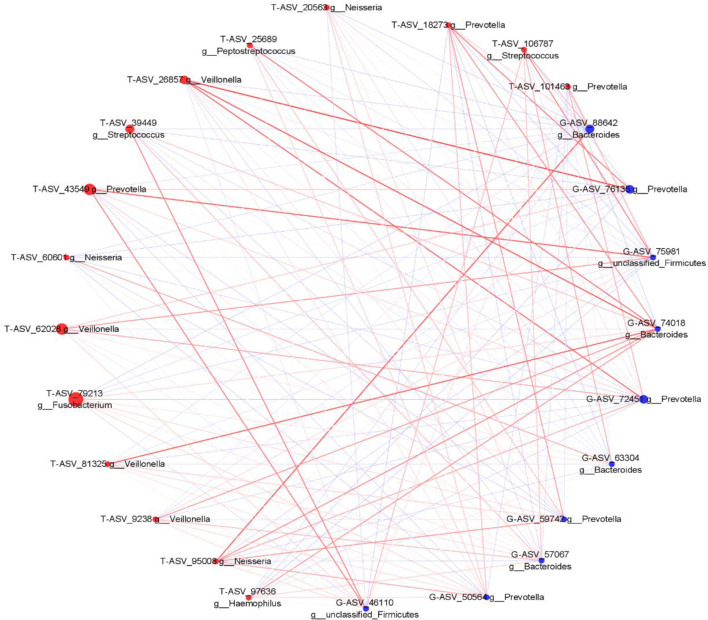
The associated network diagrams of typical tongue coating after sleep and typical intestinal microbiota (red is the tongue coating microbiota, blue is the intestinal microbiota, the size of the circle represents the abundance of the flora, the larger the area of the circle, the higher the abundance; the red line represents the positive correlation, the blue line represents the negative correlation, the thicker the line, the higher the correlation coefficient).

We analyzed the proportion of the typical oral microbiota in the fecal microbiota, and the proportion of the typical fecal microbiota in the oral microbiota before and after sleep. The results showed that the oral typical bacteria Prevotella had the highest proportion in the intestinal microbiota, which was 28.25%. Prevotella, the typical intestinal bacteria, also has the highest proportion in the oral microbiota, which was 21.1% before sleep and 30.75% after waking up (**Supplementary Figures 5, 6**). It is suggested that Prevotella is a common genus shared between the oral microbiota and intestine. Through Spearman correlation analysis, we found that the correlation coefficient of Prevotella was 0.322 (p < 0.05) between the tongue coating before sleep and intestine, and the correlation after waking was not statistically significant. The correlation coefficient between Veillonella in the oral microbiota and Bacteroides in the intestine was the highest, which was 0.468 (p < 0.01). Bacteroides in the intestine have the most associated microbiota in the oral microbiota (**Supplementary Figures 7, 8**). Meanwhile, we constructed the co-abundance network of tongue coating and intestinal microbiota before and after sleep, which visually demonstrated the relationship between the oral and intestinal microbiota (**Figures 9**, **10**).

**Figure 11:**
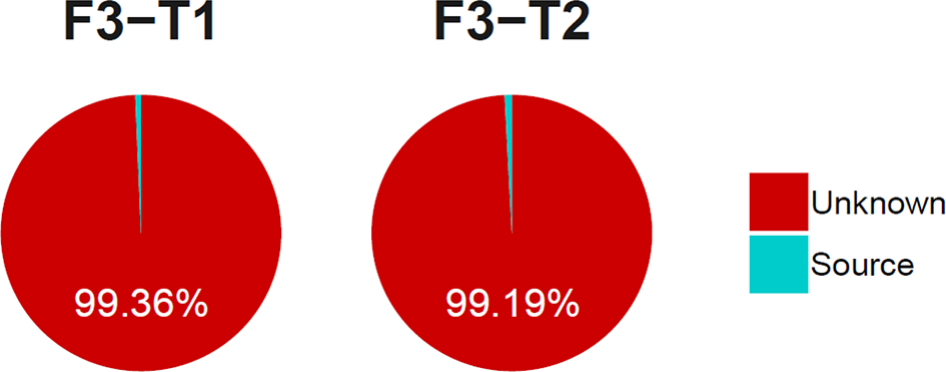
The proportion of the tongue coating microbiota before and after sleep colonized in intestine. (T1 and T2 are the tongue coating microbiota before and after sleep respectively, F3 is the intestinal microbiota and the source suggests it is derived from the tongue coating microbiota).

There were shared bacteria that existed in the oral and intestine, and they had the co-abundance changes. To clarify whether there was ectopic colonization of oral microbiota in the intestinal of healthy people, we used the SourceTracker method to trace the source of the intestinal microbiota. The results showed that the proportion of the intestinal microbiota derived from the oral microbiota was less than 1% (**Figure 11**). It indicated that oral microbiota was difficult to colonize in the intestine, which was consistent with another research (Atarashi et al., 2017), and it would provide reference for related studies in diseases.

## Discussion

There are circadian rhythmic changes in oral microbiota. The microbial genus, which accounts for 68.4%–89.6% of the total abundance in saliva, has been observed to oscillate markedly within 24 h. These periodic changes of the microorganisms enrich the functions of environmental responses such as various transporters and two-component regulatory systems at night and the metabolic functions of vitamin and fatty acid biosynthesis in the morning (Takayasu et al., 2017). With the in-depth development of microbiome and microbial metabolomics in disease research, flora and its metabolites are no longer biomarkers of pathological changes in the body, but have also become targets for disease intervention and treatment (Rosier et al., 2018; Panza et al., 2019; Sorgdrager et al., 2019). At present, probiotics, prebiotics, and synbiotics are making rapid progress in disease prevention and treatment (Vespasiani-Gentilucci et al., 2018; Vasquez et al., 2019; Li et al., 2020). Studying the circadian rhythm of microorganisms under physiological conditions will provide an important reference for determining the timing of microbial therapy.

The results of this study showed that the mainly circadian changes in the tongue coating microorganisms are the changes of the diversity and the abundance. The diversity and the number of ASV of the tongue coating microbiota after waking up were higher than those before sleep, but there was no statistical difference in the diversity. However, there were obvious differences on the abundance between them. Especially the abundance of Alloprevotella had the most obvious changes, which was significantly higher after waking up than before going to bed and which belonged to the Bacteroidetes phylum. Related studies had found the changing trend of Bacteroidetes at different time points in individuals. Although there were obvious individual differences in abundance values, the overall abundance showed obvious diurnal abundance changes (Takayasu et al., 2017), which were consistent with our results. Studies had found that oral Bacteroidetes was related to type 2 diabetes mellitus (Chen et al., 2020), inflammatory bowel disease (IBD) (Said et al., 2014), reflux esophagitis (Liang et al., 2021), etc. The findings of this study may provide some references for establishing the best microflora therapeutic intervention time for these diseases.

In terms of the relationships between the oral cavity and the intestinal flora, we found that the diversity and richness of the intestinal microbiota were significantly higher than those of the oral. Bacteroidetes, Firmicutes, and Proteobacteria were the main microbiota shared by the tongue coating and intestine (phylum level), but there were big differences in the microbiota structure of the two sites and they had different dominant microbiota distributions. By analyzing the distribution of typical bacterial genera, we found that Prevotella was a common shared bacterial genus in the oral cavity and intestine, and the relative abundance of Prevotella between the two sites was positively correlated. Bacteroides in the intestine had the most bacteria associated with the oral cavity and had the highest correlation coefficient with Veillonella in the oral cavity, which was 0.468 (p < 0.01). These results indicated that the abundance of some microbiota in the oral cavity and the intestine had a common changing trend.

Prevotella can improve glucose metabolism and insulin sensitivity through mechanisms related to the fermentation of dietary fiber to produce succinate (Kovatcheva-Datchary et al., 2015; De Vadder et al., 2016). Meanwhile, Prevotella and Bacteroides in the intestine have been identified as the main species that drive the link between branched-chain amino acid (BCAA) biosynthesis and insulin resistance (Pedersen et al., 2016). These investigations identified how specific bacteria might be linked to nutrients such as amino acids, making it possible for the gut microbiota to be a choice for the development of preventive interventions or therapeutic options (Cani, 2018). However, relationships between the oral and intestinal microbiota found in this study might provide references for disease monitoring, microbial treatment, and medicament intake time.

Veillonella and Bacteroides are the main bacteria in the tongue coating and intestinal tract, respectively. Veillonella, a Gram-negative bacterium, is a strictly anaerobic bacterium and is considered to be one of the early oral biofilm colonizers, associated with opportunistic infections and halitosis. The bacterium is also distributed in the human gastrointestinal tract (Mergenhagen, 1965; Gronow et al., 2010). At present, Veillonella can be divided into 11 species, and there are differences in the distribution of Veillonella species in the healthy young population (Mashima et al., 2011). Bacteroides are also gram-negative obligate anaerobic bacteria that construct the majority of gut bacteria (Kim et al., 2017). It is usually beneficial in the gut, and it is a major player in the maintenance of the gut microbial food web (Zafar, 2021). This study found that Veillonella in tongue coating was positively correlated with Bacteroides in the intestinal tract, and it was statistically significant. It is suggested that there is a common change trend between them, and it may be possible to predict the change of Bacteroides in the intestine by detecting the change of the abundance of Veillonella in the tongue coating. However, the 16sRNA technology used in this study cannot be accurate to the species level. Identifying the specific relationship between the tongue coating and intestine at the species level can provide a basis for subsequent verification studies.

There are differences in the composition of the intestinal flora in different diseases (Chen et al., 2016; Liu et al., 2020; Wu, 2020). Exploring the connections between oral and intestinal microbiota can make the microbiota intervention and disease monitoring more convenient. Majority of previous studies have shown that the ecological imbalance of the oral microbiota may be related to the imbalance of intestinal microecology (Said et al., 2014). The increase of oral-derived microbiota in the intestine can lead to the occurrence of diseases (Atarashi et al., 2017), such as inflammatory bowel disease (IBD) (Gevers et al., 2014) and liver cirrhosis (Chen et al., 2016). However, these studies only found that different disease-related bacteria in the intestine commonly existed in the oral cavity, and it did not confirm whether these bacteria were ectopic colonization from the oral microbiota. This study found that there were some coexisting microbiota distributed in the oral microbiota and intestine in healthy people. For example, Prevotella accounts for 28.25% in the intestinal microbiota, 21.1% before sleep and 30.75% after waking up in the oral microbiota. However, the rate of oral bacteria colonizing in the intestines was less than 1% in healthy people. This result suggested that common bacteria coexisting in the oral cavity and intestines might not colonize the intestinal tract by the oral cavity.

Ectopic colonization of oral microbiota is associated with diseases. Most of the patient-enriched gut microbiota in liver cirrhosis come from the oral cavity, such as Veillonella and Streptococcus. The pathogens Campylobacter and Haemophilus parainfluenzae may also invade the intestine through the oral route, possibly *via* contaminated food. This suggested that the cirrhosis status was related to the ectopic colonization of the oral flora in the intestine. This exogenous invasion may occur not only in the colon but also in the ileum, promoting the over-proliferation of small intestinal bacteria associated with liver cirrhosis (Qin et al., 2014). Animal experiments showed that bacteria from the human gut microbiota were unable to acquire niches in the mouse gut containing the autochthonous microbiota, although the mouse gut is highly selective, within the fundamental niche of bacterial phylotypes derived from various environments (Seedorf et al., 2014). In the digestive tract of human oral microbiota-associated mice, the number of OTUs gradually decreased from the stomach to the distal intestine. In the cohoused mice, oral bacteria were more likely to ectopically colonize the small intestine than the distal intestine (Li et al., 2019). In this study, stool samples from healthy volunteers only represented the microbiota of the lower gastrointestinal tract (Human Microbiome Project Consortium, 2012). Due to study limitations, we could not analyze the bacterial composition of different parts of the gut. Therefore, further studies are needed to clarify the general and specific conditions of ectopic colonization of oral flora in the gut in healthy people.

This study found that the circadian changes of tongue coating microorganisms in healthy people were mainly manifested on changes in abundance, and the function of these microbiota in the oral still needs further research. There was identical microbiota existing in the oral microbiota and intestine of healthy people. Even though the proportion of oral microbiota colonizing in the intestine was low, there was a correlation between the abundance of the same microbiota in the two sites. Due to the limitations of the SourceTracker method, we were currently unable to clarify the specific bacteria of the intestinal flora derived from the oral cavity. Meanwhile, this study was a pilot study (In, 2017) with little sample size, and the subjects in this study were healthy college students whose age were young. Hence, exploring further data analysis methods and designing the research which include different ages, diseases, and multiple time points in future would identify the relationship between oral and intestine microbiota more clearly. However, the connection between the oral and intestinal flora found in this study suggests the necessity of combining the two-site flora to conduct simultaneous research in future disease and drug research.

## Conclusions

The circadian changes of tongue coating microorganisms in healthy individuals mainly manifest on the diversity and their abundance; this may provide some references for the study on medication time. The tongue coating and intestine have a shared distribution of microbiota, and their abundance has correlations which can be affected by the circadian changes of tongue coating microorganisms. In healthy people, the colonized proportion of tongue coating bacteria in the intestine is very low, and how it changes in diseases remains to be further studied. The study on the relationship between the tongue coating and the intestinal microbiota can provide a reference for disease monitoring, microbiota treatment, and the development of Chinese tongue diagnostic technology.

## Data Availability Statement

The original contributions presented in the study are publicly available in NCBI under accession number PRJNA782768.

## Ethics Statement

The studies involving human participants were reviewed and approved by the ethics committee of Shuguang Hospital Affiliated to Shanghai University of TCM. The ethics committee number is 2018-626-55-01, and the clinical trial registration number is ChiCTR1900026008. The participants provided their written informed consent to participate in this study.

## Author Contributions

Jiang T and Guo XJ contributed equally to this work and participated in the experimental research design, case collection, data processing, and paper writing. Huang JB, Hu XJ, Shi YL, li J, and Cui LT participated in methodological guidance. Ma XX, Yao XH, and Cui J participated in research design guidance and paper revision. Lou JD, Liang MC, Fu HY, and Liu JY participated in the sample collection. Guo ZL and Yuan P participated in the data collation. Xu JT and Tu LP participated in the design and implementation of the research and provided important guidance for data processing, writing, and revision of the paper. All authors contributed to the article and approved the submitted version.

## Funding

This study was supported by the National Key Technology R&D Program of China [grant numbers 2017YFC1703301], the National Natural Science Foundation of China [grant numbers 81873235, 82104736, 82104738], Shanghai Science and Technology Commission [21010504400], and Shanghai Municipal Commission [grant numbers 201940117, 2020JQ003].

## Conflict of Interest

The authors declare that the research was conducted in the absence of any commercial or financial relationships that could be construed as a potential conflict of interest.

## Publisher’s Note

All claims expressed in this article are solely those of the authors and do not necessarily represent those of their affiliated organizations, or those of the publisher, the editors and the reviewers. Any product that may be evaluated in this article, or claim that may be made by its manufacturer, is not guaranteed or endorsed by the publisher.
