# Supplementary material for: Relationships Between Diurnal Changes of Tongue Coating Microbiota and Intestinal Microbiota
Source: Front Cell Infect Microbiol. 2022 Mar 31;12:813790. doi: 10.3389/fcimb.2022.813790 (PMC9008461; doi:10.3389/fcimb.2022.813790)
Supplement: Supplementary file 1 [file Presentation_1.pdf]

Supplementary Material

1. Supplementary Figures and Tables

1.1 Supplementary Figures

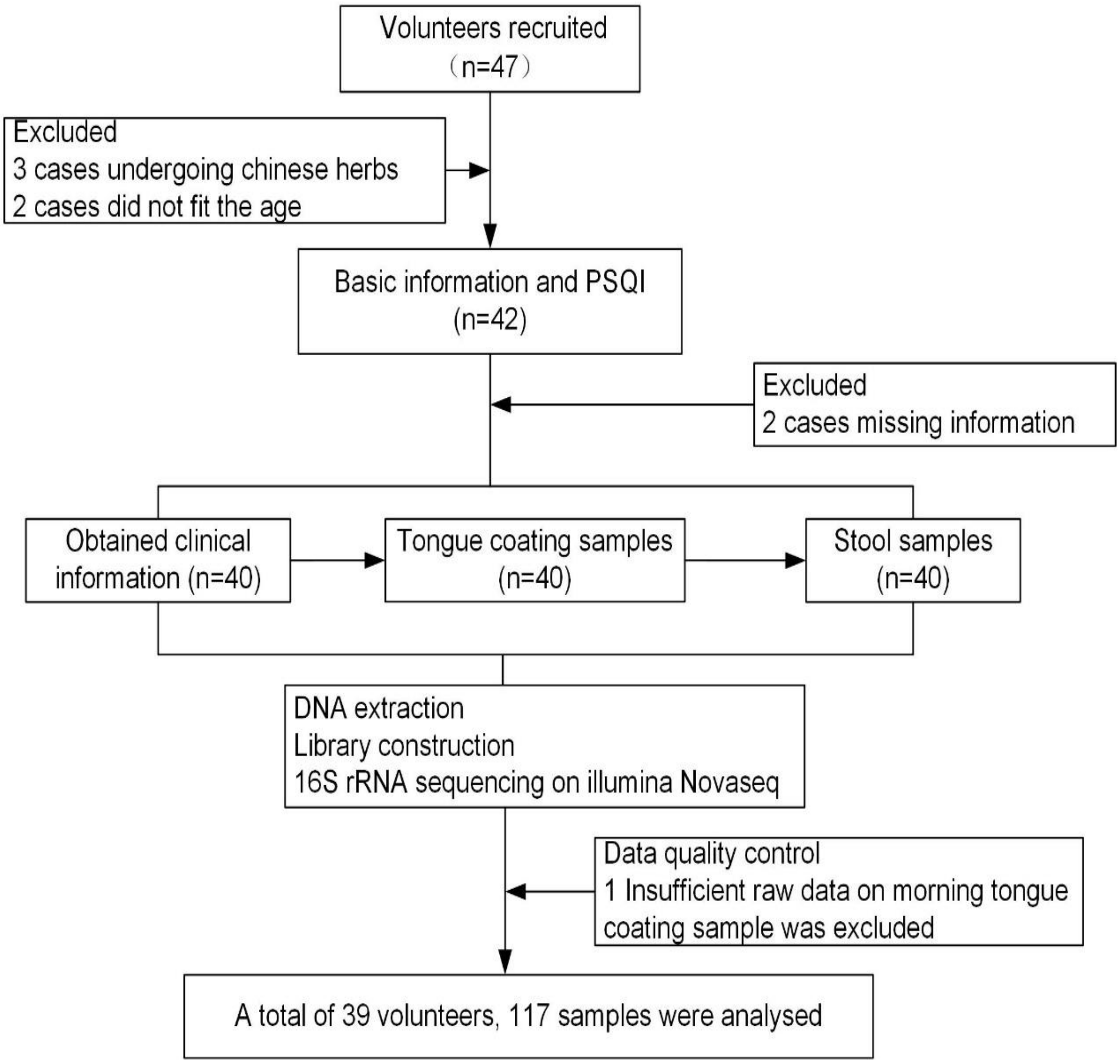

Supplementary Figure 1. The research processes

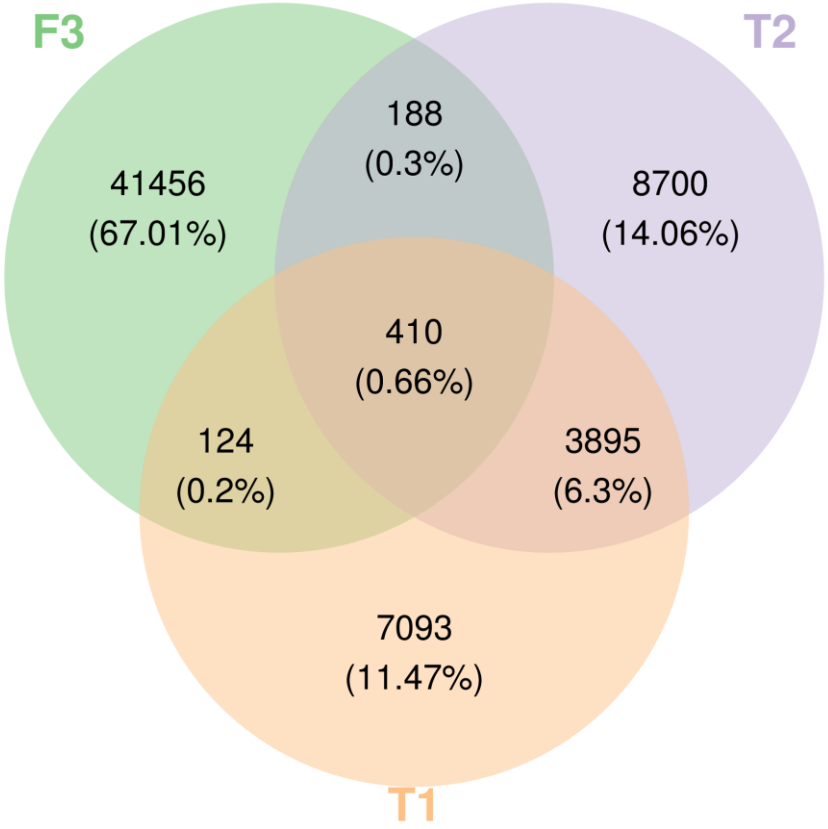

Supplementary Figure 2. The Venn diagram of the ASV numbers of the tongue coating and intestinal microbiota (the composition ratio in parentheses， T1 and T2 are the tongue coating microbiota before and after sleep respectively, F3 is the intestinal microbiota)

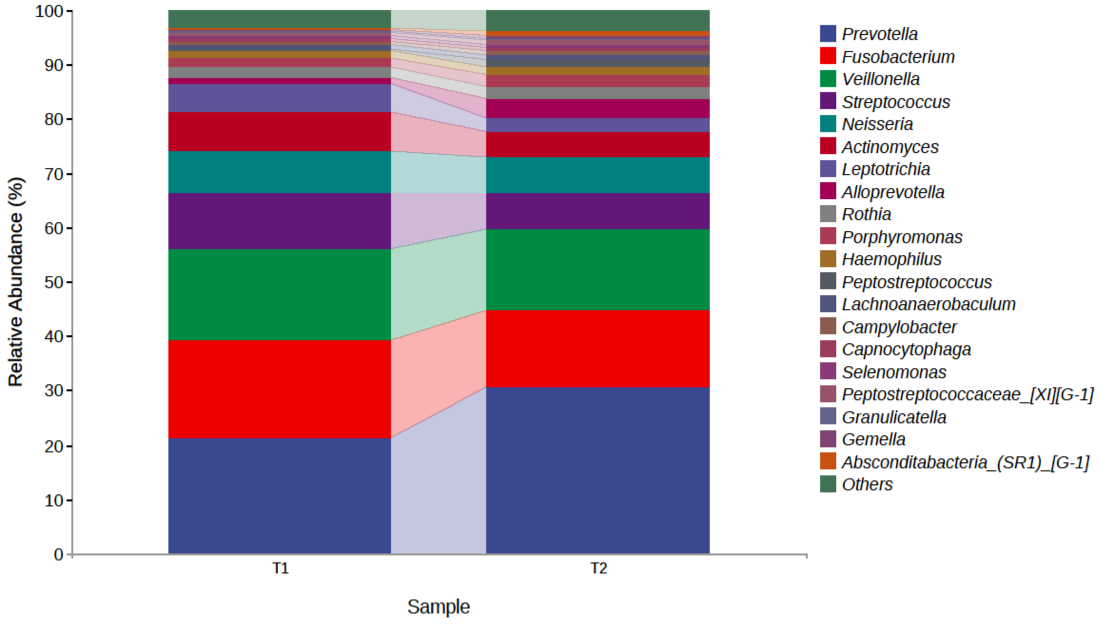

Supplementary Figure 3. Compositions and relative abundance of the bacterial community in both groups at the genus level (top 20 genera，T1 and T2 are the tongue coating microbiota before and after sleep respectively)**.**

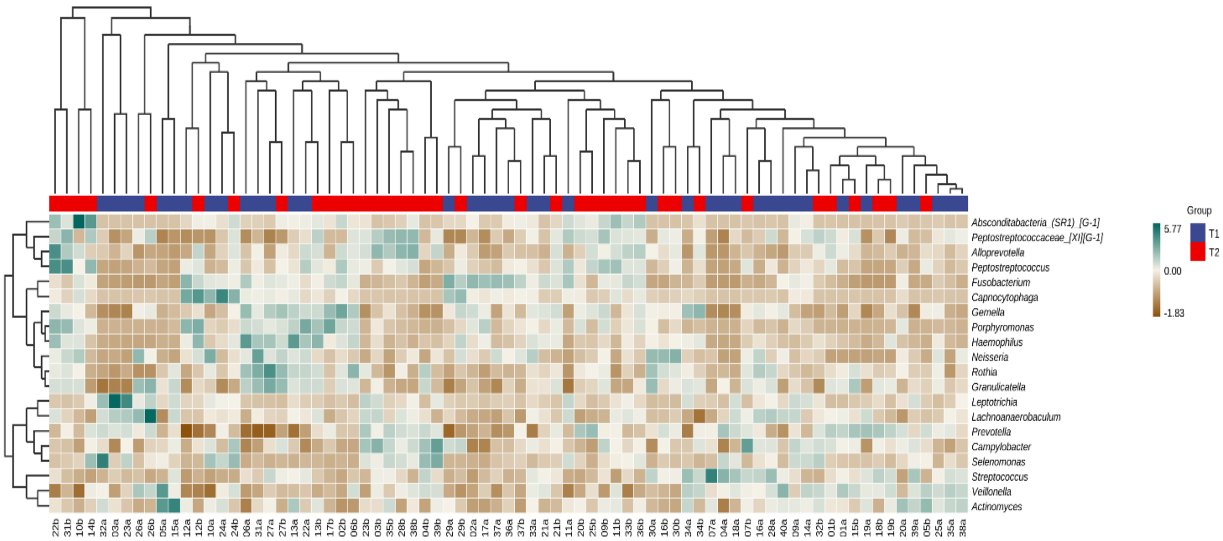

Supplementary Figure 4. Compositions and relative abundance of the bacterial community in both groups at the genus level (top 20 genera).

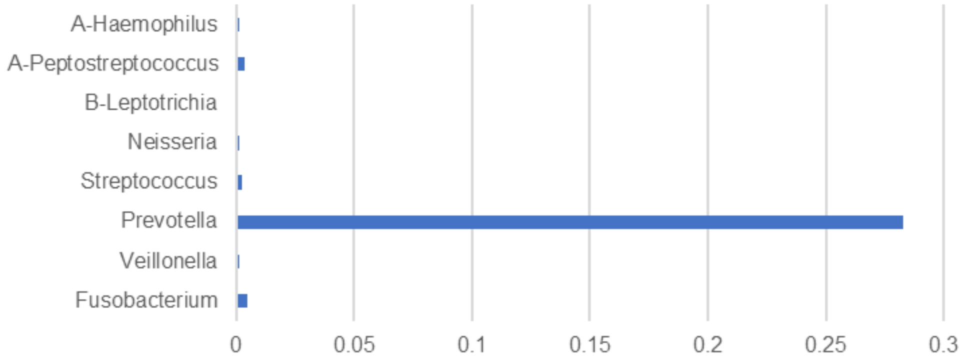

Supplementary Figure 5. The porpotion of the typical oral microbiota in the intestine.

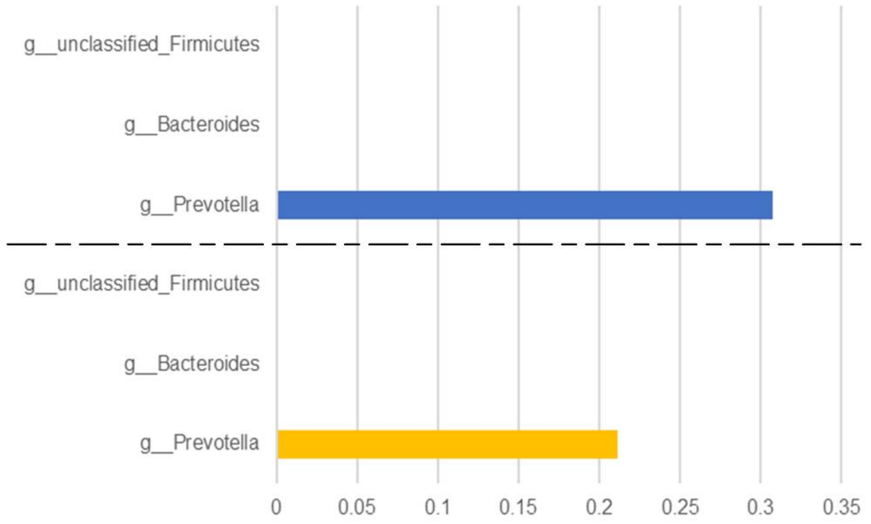

Supplementary Figure 6. The porpotion of the typical intestinal microbiota in the tongue coating before and after sleep respectively (Corresponding to below and above the black dotted line respectively).

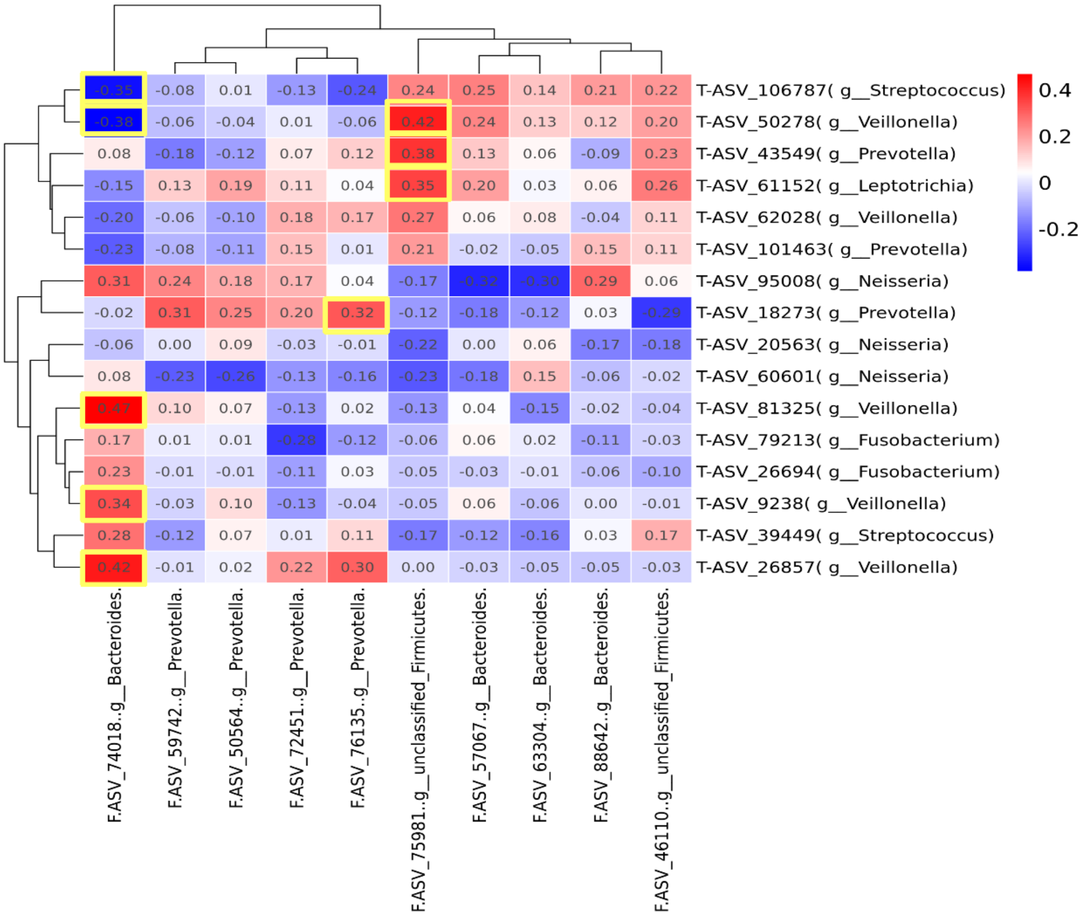

Supplementary Figure 7. The correlation heat maps of typical tongue coating before sleep and typical intestinal microbiota. (yellow box indicates that the correlation between the two is statistically significant, P<0.05, vertical axis Is the tongue coating microbiota, and the horizontal axis is the intestinal microbiota).

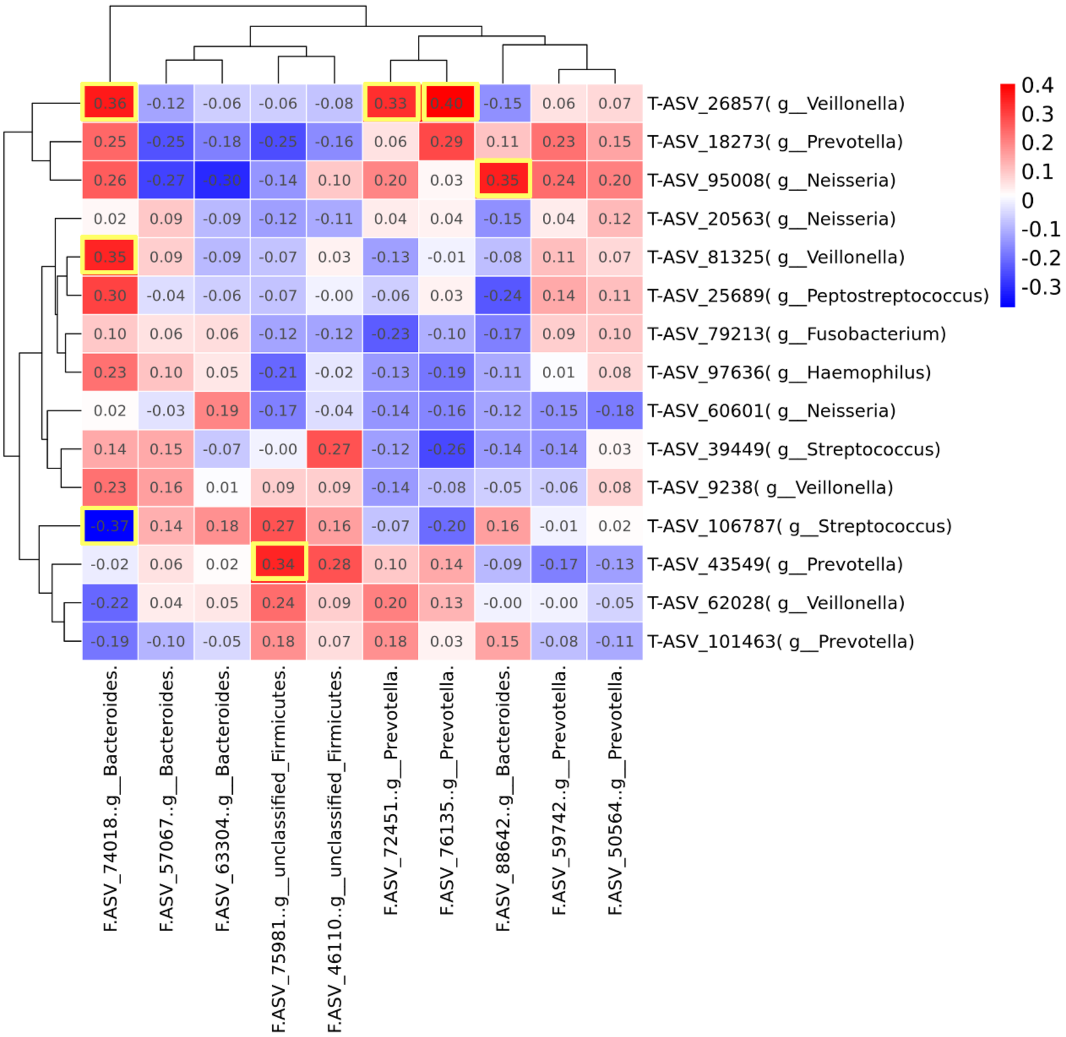

Supplementary Figure 8. The correlation heat maps of typical tongue coating after sleep and typical intestinal microbiota respectively (yellow box indicates that the correlation between the two is statistically significant, P<0.05, vertical axis Is the tongue coating microbiota, and the horizontal axis is the intestinal microbiota).

1.2 Supplementary tables

Supplementary Table 1 Basic information of volunteers

Demographics, clinical parameters, and habits	Participants
Sex	9males; 30females
Age	21.35±2.66
BMI	20.63±4.54
Blood pressure:<br>Systolic blood pressure;<br>Diastolic blood pressure;	108.77±11.46/72.25±8.00
Dietary structure:<br>1.Mainly Carbohydrate;<br>2.Mainly meat and fatty foods;<br>3.A balanced diet	N1=25; N2=1; N3=13
Taste hobby: 1.Sweet; 2.Spicy; 3.Normal.	N1=6; N2=14; N3=15;<br>N1+2=4
Dentures/missing teeth	N=0
Periodontal diagnosis	N=0
Intraoral applications	N=0
Daily medication use	N=0
Recreational drug use	N=0
History of chronic disease	N=0
Antibiotic use in past 3 months	N=0
Smokers	N=0
Drinkers	N=0
PSQI score	4.31±2.20

Supplementary Table 2 Statistical table of analysis of differences in the distribution of tongue coating microbiota before and after sleep

Group1	Group2	Sample size	Permutations	Pseudo F	p value	q value
all	-	78	999	2.460838	0.001	-
T1	T2	78	999	2.460838	0.001	0.001

Supplementary Table 3 The difference of tongue coating microbiota before and after sleep (Top 10 genus）

Group	Prevotella	Fusobacterium	Veillonella	Streptococcus	Neisseria	Actinomyces	Leptotrichia	Alloprevotella	Rothia	Porphyromonas
T1	0.212±0.125	0.179±0.191	0.169±0.092	0.103±0.088	0.077±0.085	0.071±0.05	0.053±0.078	0.011±0.009	0.021±0.022	0.015±0.022
T2	0.307±0.115	0.145±0.121	0.15±0.07	0.066±0.045	0.064±0.066	0.048±0.027	0.023±0.029	0.036±0.023	0.021±0.016	0.022±0.027
Z	-4.535	-1.102	-1.703	-3.879	-0.949	-3.628	-3.935	-5.442	-0.326	-3.824
P	0.000	0.270	0.089	0.000	0.343	0.000	0.000	0.000	0.744	0.000

Supplementary Table 3 The difference of tongue coating microbiota before and after sleep (Top 11-19 genus）

Group	Haemophilus	Peptostreptococcus	Lachnoanaerobaculum	Capnocytophaga	Campylobacter	Peptostreptococcaceae_[XI][G-1]	Selenomonas	Granulicatella	Gemella
T1	0.014±0.019	0.005±0.005	0.008±0.004	0.007±0.012	0.005±0.003	0.004±0.003	0.005±0.008	0.004±0.003	0.004±0.003
T2	0.015±0.015	0.013±0.011	0.007±0.005	0.006±0.011	0.008±0.005	0.008±0.004	0.006±0.007	0.005±0.003	0.005±0.003
Z	-1.689	-5.428	-1.716	-0.355	-3.586	-4.982	-0.963	-1.423	-3.684
P	0.091	0.000	0.086	0.723	0.000	0.000	0.336	0.155	0.000

Supplementary Table 4 The difference of typical tongue coating microbiota before and after sleep (genus level)

Group	Alloprevotella	Peptostreptococcus	Peptostreptococcaceae_[XI][G-1]	Proteus	Bacteroides	Actinomyces	Streptococcus	Leptotrichia	Campylobacter	Lachnoanaerobaculum
T1	0.011±0.009	0.005±0.005	0.004±0.003	0.0002±0.0001	0.0004±0.0002	0.072±0.050	0.103±0.088	0.053±0.079	0.004±0.003	0.008±0.004
T2	0.036±0.023	0.013±0.011	0.008±0.004	0.0002±0.0001	0.0007±0.0004	0.048±0.027	0.066±0.045	0.023±0.029	0.008±0.005	0.007±0.005
Z	-5.442	-5.428	-4.982	-2.665	-3.642	-3.628	-3.879	-3.935	-3.586	-1.716
P	0.000	0.000	0.000	0.008	0.000	0.000	0.000	0.000	0.000	0.086
